# Supplementary figures and images for: Predicting the potential distributions of the invasive cycad scale Aulacaspis yasumatsui (Hemiptera: Diaspididae) under different climate change scenarios and the implications for management
Source: PeerJ. 2018 May 23;6:e4832. doi: 10.7717/peerj.4832 (PMC5970564; doi:10.7717/peerj.4832)

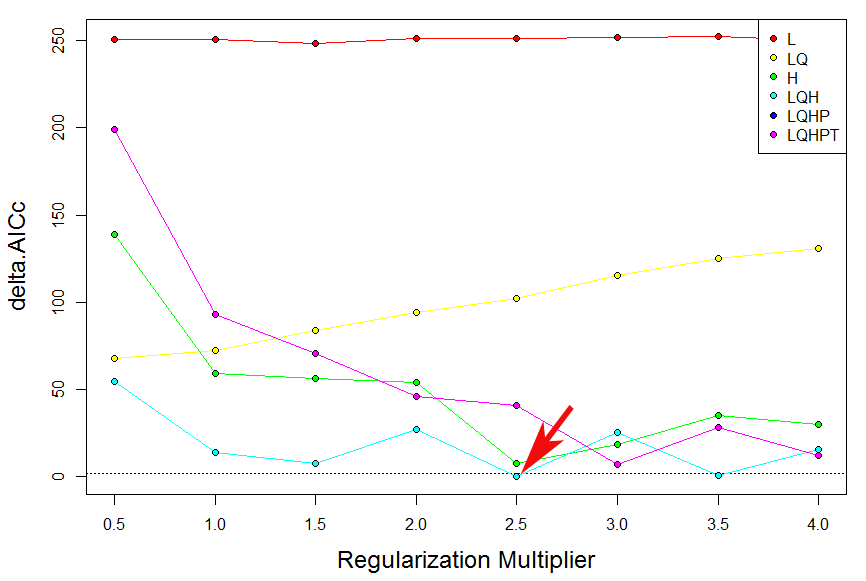

Supplement: Figure S1 — Red arrow indicates the delta AICc-chosen setting. L = Linear;Q = Quadratic; H = Hinge; P = Product; T = Threshold. [file peerj-06-4832-s001.png]

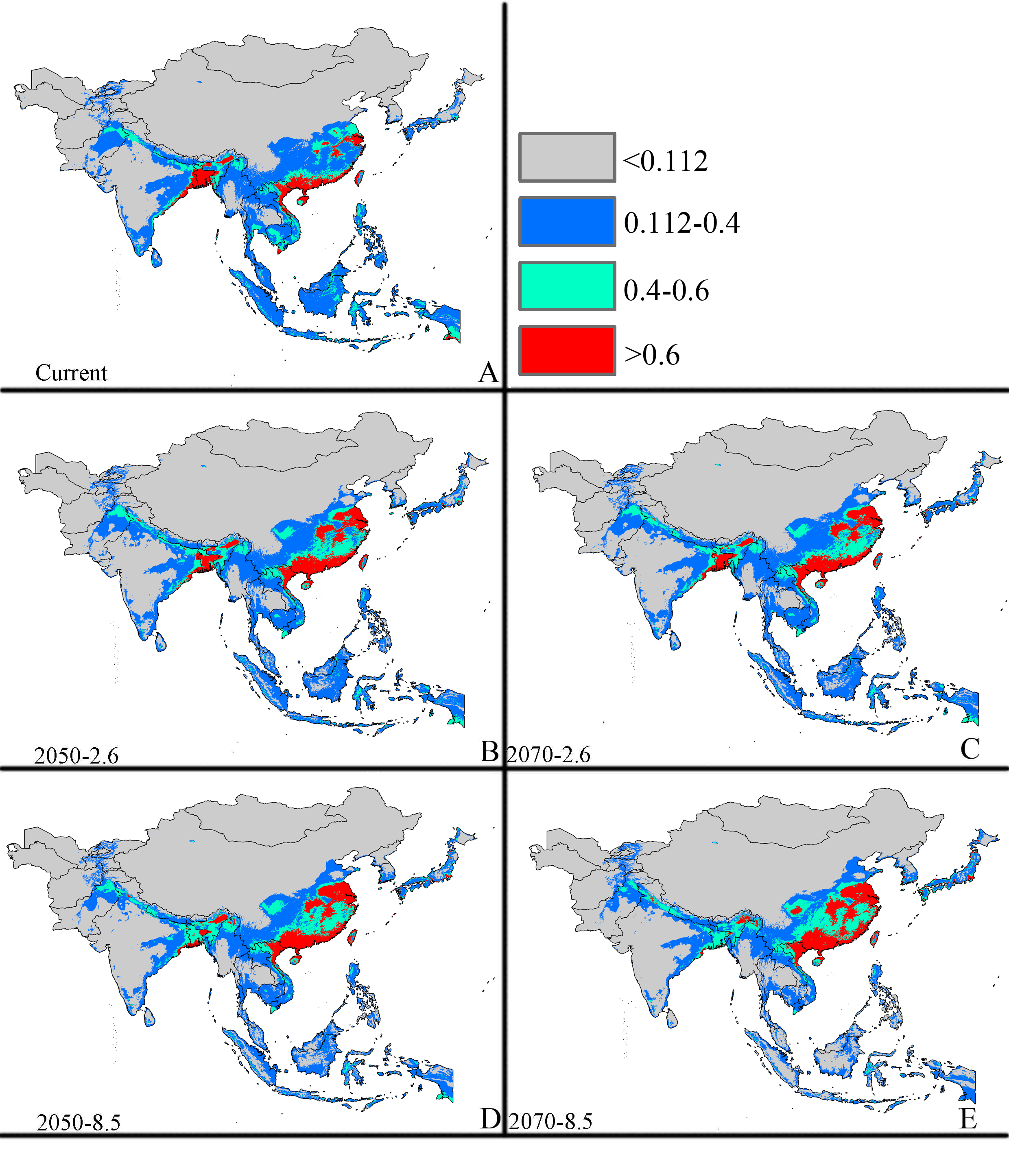

Supplement: Figure S2 — The letters show predictions for the current, RCP 2050-2.6.RCP 2050-8.5, RCP 2070-2.6, RCP 2070-8.5 (A: Current; B: RCP 2050-2.6; C: RCP 2070-2.6, D: RCP 2050-8.5; E: RCP 2070-8.5). Gray = unsuitable habitat area; Blue = low habitat suitability area; Green = moderate habitat suitability area; Red = highly habitat suitability area. The base map was created with Natural Earth Dataset (http://www.naturalearthdata.com/). [file peerj-06-4832-s002.png]

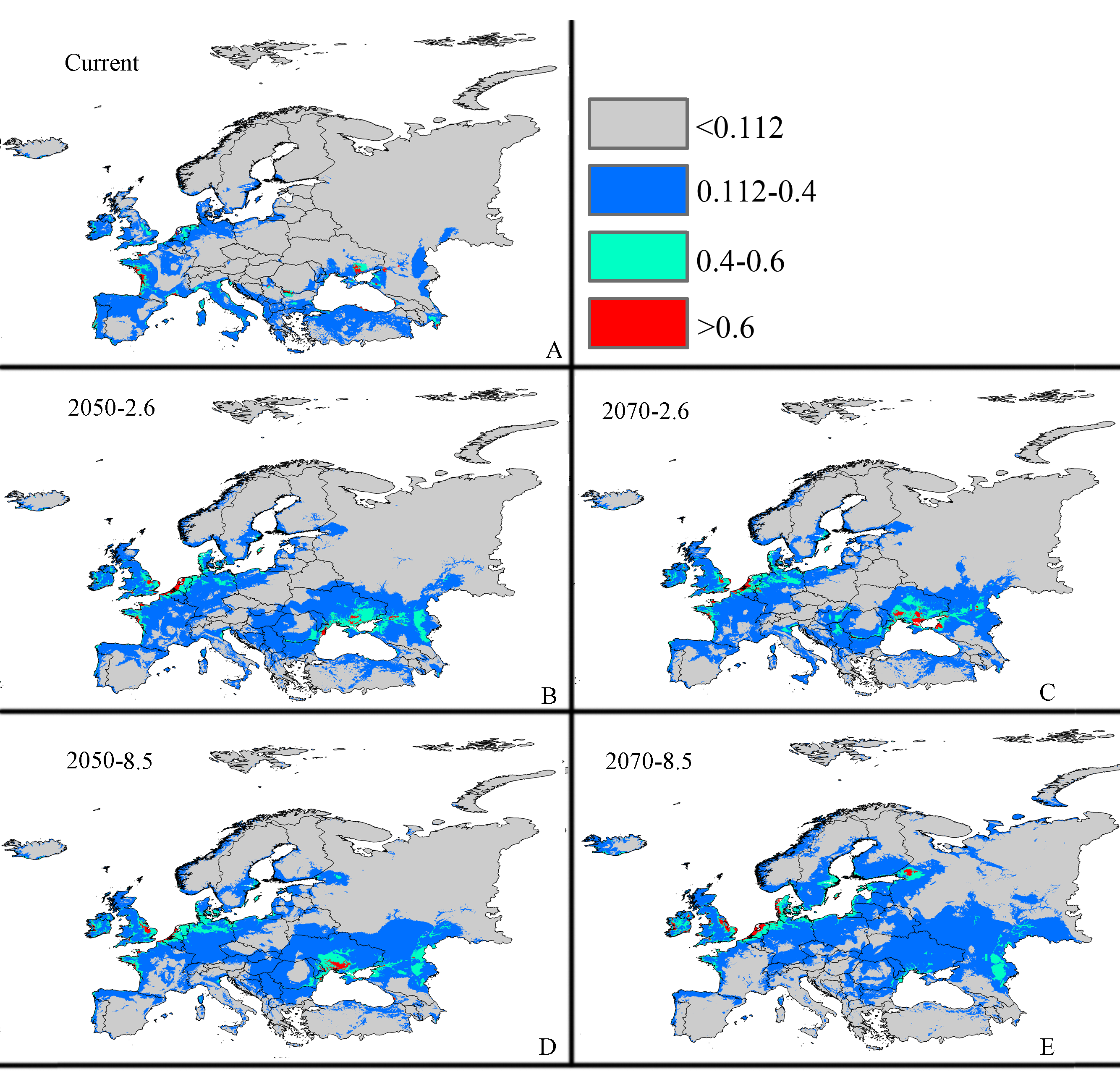

Supplement: Figure S3 — The letters show predictions for the current, RCP 2050-2.6.RCP 2050-8.5, RCP 2070-2.6, RCP 2070-8.5 (A: Current; B: RCP 2050-2.6; C: RCP 2070-2.6, D: RCP 2050-8.5; E: RCP 2070-8.5). Gray = unsuitable habitat area; Blue = low habitat suitability area; Green = moderate habitat suitability area; Red = highly habitat suitability area. The base map was created with Natural Earth Dataset (http://www.naturalearthdata.com/). [file peerj-06-4832-s003.png]

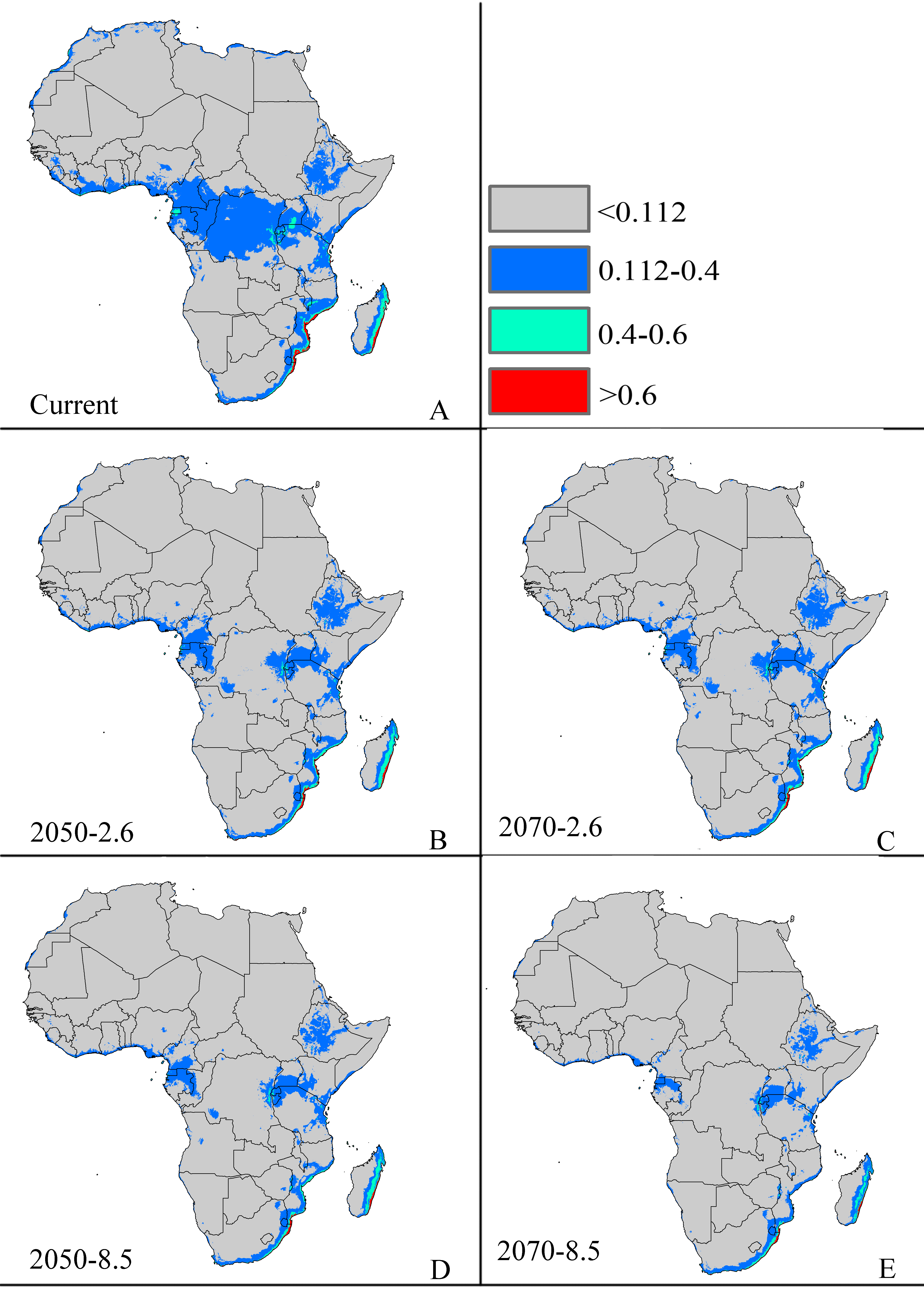

Supplement: Figure S4 — The letters show predictions for the current, RCP 2050-2.6.RCP 2050-8.5, RCP 2070-2.6, RCP 2070-8.5 (A: Current; B: RCP 2050-2.6; C: RCP 2070-2.6, D: RCP 2050-8.5; E: RCP 2070-8.5). Gray = unsuitable habitat area; Blue = low habitat suitability area; Green = moderate habitat suitability area; Red = highly habitat suitability area. The base map was created with Natural Earth Dataset (http://www.naturalearthdata.com/). [file peerj-06-4832-s004.png]

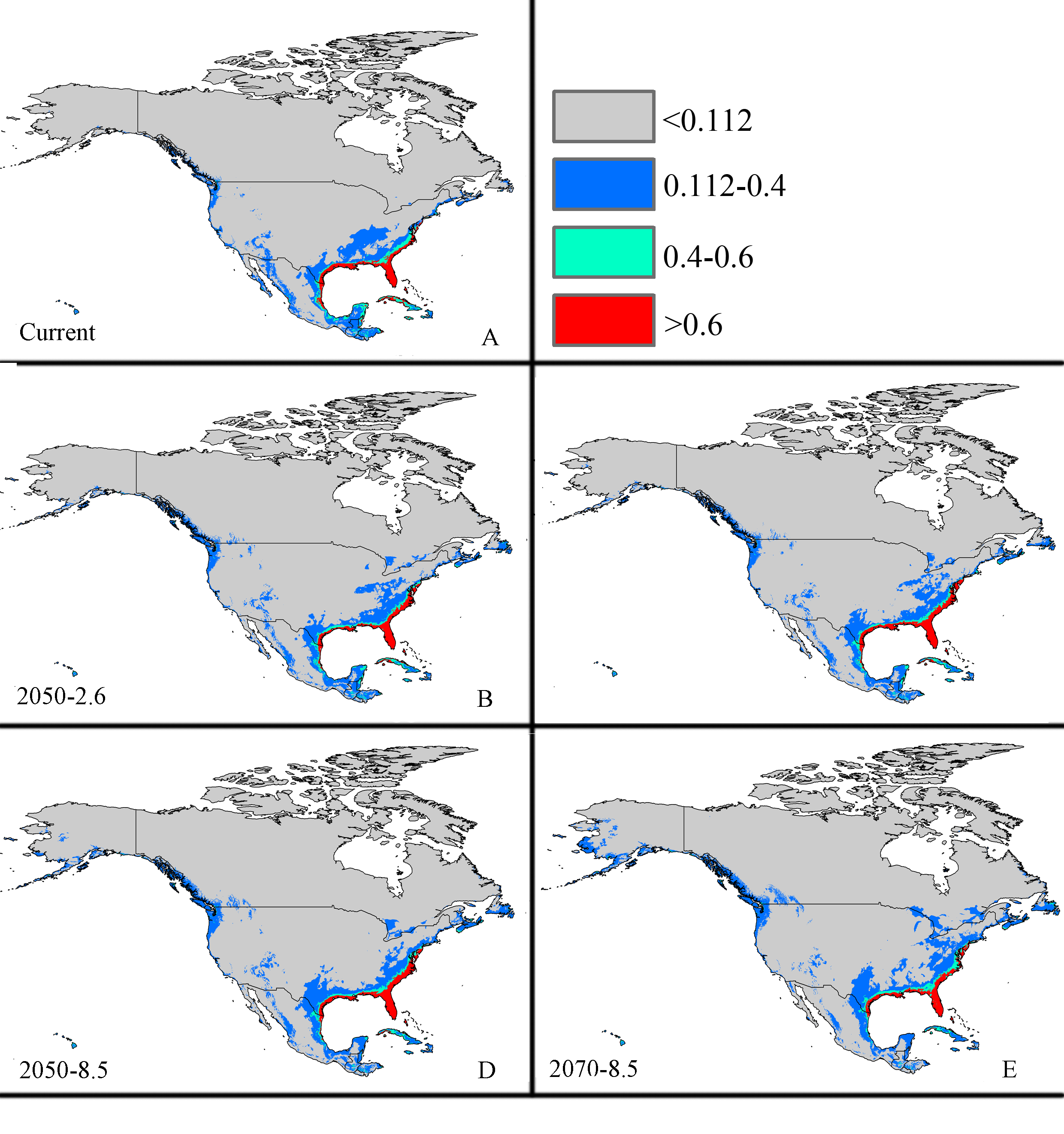

Supplement: Figure S5 [file peerj-06-4832-s005.png]
