# Supplementary material for: Predicting the potential distributions of the invasive cycad scale Aulacaspis yasumatsui (Hemiptera: Diaspididae) under different climate change scenarios and the implications for management
Source: PeerJ. 2018 May 23;6:e4832. doi: 10.7717/peerj.4832 (PMC5970564; doi:10.7717/peerj.4832)
Supplement: Table S1 [file peerj-06-4832-s006.docx]

**Table S1 References used to compile the dataset**

**Cave RD. 2006.** Biological control agents of the cycad Aulacaspis scale, *Aulacaspis yasumatsui*. *Proceedings of the Florida State Horticultural Society* **119:** 422-424

**Cave RD, Nguyen R, Manrique V, Avery PB. 2009.** New research on two natural enemies of the cycad aulacaspis scale. *The Cycad Newsletter* **32:** 22-23

**Emshousen C, Mannion C, Glenn H. 2004.** Management of cycad Aulacaspis scale, *Aulacaspis yasumatsui* Takagi. *Proceedings of the Florida State Horticultural Society* 117: 305-307.

**Evans GA, Dessart P, Glenn H. 2005.** Two new species of Aphanogmus (Hymenoptera: Ceraphronidae) of economic importance reared from Cybocephalus nipponicus (Coleoptera: Cybocephalidae). *Zootaxa* **1018:** 47-54.

**Flores D, Carlon J. 2009.** Fortuitous Establishment of *Rhyzobius lophanthae* (Coleoptera: Coccinellidae) and *Aphytis lingnanesis* (Hymenoptera: Encyrtidae) in South Texas on the Cycad Aulacaspis scale, *Aulacaspis yasumatsui* (Hemiptera: Diaspididae). *Southwestern Entomologist Scientific Note* **34(4):** 489-492.

**Germain JF, Hodges GS. 2007.** First report of Aulacaspis yasumatsui (Hemiptera: Diaspididae) in Africa (Ivory Coast), and update on Distribution. *Florida Entomologist* **90(4):** 755-756

**González-Gómez R, Riverón-Giró FB, García-González A, Martinez-Rosas RM, Solís-Montero L. 2016.** First report of Aulacaspis yasumatsui (Hemiptera: Diaspididae) in Mexico. *Florida Entomologist* **99(3):** 583-584.

**Howard FW, Hamon A, Mclaughlin M, Weissling T, Yang SL. 1999.** *Aulacaspis yasumatsui* (Hemiptera: Sternorryncha: Diaspididae). A scale insect pest of cycads recently introduced in to Floria. *The Florida Entomologist* **82(1):** 14-26

**Howard FW, Weissling TJ.** **1999.** Questions and answers about the cycad aulacaspis scale insect. *Proceedings of the Florida State Horticultural Society* **112:** 243-245.

**Kozar F, Benedicty ZK, Fetyko K, Kiss B, Szita E. 2013.** An annotated update of the scale insect checklist of Hungary (Hemiptera, Coccoidea). *Zookeys* **309:** 49-66.

**Lindstrom AJ, Hill KD, Stanberg LC. 2009.** The genus Cycas (Cycadaceae) in Indonesia. *Telopea* **12(3):** 385-418.

**Luo YT, Xu YF. 2011.** Biological study on the natural parasitoids of cycad scale *Aulacaspis yasumatsui*. Taiwan Province Agriculture and Forestry Bureau Forest Bureau conservation research series.100-39, 55p.

**Marler TE, Miller R, Moore A.** **2013.** Vertical stratification of predation on Aulacaspis yasumatsui infesting cycas micronesica seedings. *HortScience* **48(1):** 60-62.

**Marler TE.** 2013. Temporal variations in Leaf Miner, Butterfly, and Stem Borer Infestations of *Cycas micronesica* in relation to *Aulacaspis yasumatsui* incidence. *HortScience* 48(10): 1334-1338.

**Marler TE, Lawrence JH.** **2012.** Demography of Cycas micronesica on Guam following introduction of the armoured scale Aulacaspis yasumatsui. *Journal of Tropical Ecology* **28:** 233-242.

**Marler TE, Moore A. 2010.** Cryptic scale infestations on *Cycas revolute* Facilitate scale invasions. *Hortscience* **45(5):** 837-839.

**Milek TM, Simala M, Novak. 2008.** Species of genus Aulacaspis Cockerell, 1836. (Hemiptera: Coccoidea: Diaspididae) in Croatia, with emphasis on *Aulacapsis yasumatsui* Takagi, 1977. *Entomologia Croatica* **12(1):** 55-64.

**Munlappan R, Watson GW, Evans GA, Rauf A, Ellenrieder N. 2012.** Cycad Aulacaspis scale, a newly introduced insect pest in Indonesia. *Journal of biosciences* **19(3):** 110-114.

**Normerk BB, Normark RD, Vovides A, Lislie Solis-Montero L, Gonzalez-Gomez R, Pulido-Silva MT, Escobar-Castellanos MA, Dominguez M, Perez-Farrera MA, Janda M, Cibrian-Jaramillo A. 2017.** Cycad aulacaspis scale (*Aulacaspis yasumatsui* Takagi, 1977) in Mexico and Guatemala: a threat to native cycads. *BioInvasions Records* **6(3):** 187-193.

**Pencheva A, Yovkova M. 2016.** New data on alien insect pests of ornamental plants in Bulgaria. *Forestry Ideas* **22(1):** 17-33.

**Ronald RD, Chao JT, Kumashiro B, Marler T, Moore A, Muniappan R, Watson GW. 2013.** Status and biological control of Cycad Aulacaspis scale. *Biocontrol News and Information* 34(1): 1-4.

**Smith TR. 2007.** A new species of *Cybocephalus* (Coleoptera: Cybocephalidae) from Taiwan and a new distribution record for *Cybocephalus nipponicus*. *The Coleopterists Bulletin* **61(4):** 503-508.

**Stocks IC. 2013.** Recent adventive scale insects (Hemiptera: Coccoidea) and whitefiles (Hemiptera: Aleyrodidae) into Florida and the Caribbean Basin. In: Pena JE. Ed. Potential invasive pests of Agricultural crops. CAB international, Wallingford, UK, pp342-362.

**Takagi S, De faveri S. 2009.** Notes on scale insects of *Aulacaspis* associated with mangroves and cycads (Sternorrhyncha: Coccoidea: Diaspididae). *Insecta matsumurana, Series entomology. New series* **65:** 101-129.

**Trencheva K, Trenchev G, Tomov R, Wu SA. 2010.** Non-indigenous scale insects on ornamental plants in Bulgaria and China: a survey. *Entomologia Hellenica* **19:** 114-123.

**Ülgentürk S. 2015.** An unwanted passenger on Cycas palm; *Aulacaspis yasumatsui* Takagi (Hemiptera: Diaspididae). *Turkish Bulletin of Entomology* 5(4): 195-200.

**Waltman KG, Ray CH Jr, Williams ML.** 2016. The armored scale insects (Hemiptera: Diaspididae) of Alabama, USA. *Redia* **101:** 229-231.

**Wang YP. 2016.** The control technology of scale insect. *Flower Plant & Penjing* **8.**

**Watson GW, Muniappan R, Shepard BM, Sembel DT, Rauf A, Carner GR, Benson EP.** **2014.** Sap-sucking inset records (Hemiptera: Sternorrhyncha and Thysanoptera: Thripidae) from Indonesia. Florida Entomologist, **97(4):** 1594-1597.

**Weissling TJ, Howard FW, Hamon AB.1999.** Cycad aulascaspis scale, *Aulacaspis yasumatsui* Takagi (Insecta: Homoptera: Sternorrhyncha: Diaspididae). *Florida Coop Ext Service Publication document* EENY-096.

**Yang WX, Wu WD, Jiao GL, Liu F. 2009.** Biology of *Aulacaspis yasumatsui* Takagi and its control test. *Journal of Fujian Forestry Science and Technology* **36(4):** 127-129.
