# Supplementary material for: Predicting the potential distributions of the invasive cycad scale Aulacaspis yasumatsui (Hemiptera: Diaspididae) under different climate change scenarios and the implications for management
Source: PeerJ. 2018 May 23;6:e4832. doi: 10.7717/peerj.4832 (PMC5970564; doi:10.7717/peerj.4832)
Supplement: Table S3 — The highly correlation variables (r ≥ |0.85|) in PCA are in bold. [file peerj-06-4832-s008.docx]

**Table S3** Principal components analysis (PCA) of bioclimatic variables associated with occurrence of CAS

| Variables | Description | PC1 | PC2 | PC3 |
| --- | --- | --- | --- | --- |
| Bio1 | Annual Mean Temperature | **.870** | -.346 | .272 |
| Bio2 | Mean Diurnal Range | -.249 | -.377 | -.197 |
| Bio3 | Isothermality(BIO2/BIO7) (* 100) | .575 | .102 | -.614 |
| Bio4 | Temperature Seasonality(standard deviation *100) | -.724 | -.062 | .606 |
| Bio5 | Max Temperature of Warmest Month | .381 | -.544 | .645 |
| Bio6 | Min Temperature of Coldest Month | **.942** | -.065 | -.047 |
| Bio7 | Temperature Annual Range(BIO5-BIO6) | -.747 | -.251 | .425 |
| Bio8 | Mean Temperature of Wettest Quarter | .650 | -.352 | .451 |
| Bio9 | Mean Temperature of Driest Quarter | .731 | -.163 | .016 |
| Bio10 | Mean Temperature of Warmest Quarter | .501 | -.423 | .741 |
| Bio11 | Mean Temperature of Coldest Quarter | **.932** | -.202 | -.081 |
| bio12 | Annual Precipitation | .702 | .631 | .109 |
| Bio13 | Precipitation of Wettest Month | .783 | .211 | -.078 |
| Bio14 | Precipitation of Driest Month | .112 | **.859** | .366 |
| Bio15 | Precipitation Seasonality (Coefficient of Variation) | .511 | -.605 | -.399 |
| Bio16 | Precipitation of Wettest Quarter | .784 | .282 | -.068 |
| Bio17 | Precipitation of Driest Quarter | .103 | **.856** | .374 |
| Bio18 | Precipitation of Warmest Quarter | .569 | .211 | .157 |
| Bio19 | Precipitation of Coldest Quarter | .147 | .798 | .203 |
| Alt | Altitude | -.076 | .086 | **-.835** |

Eigenvalues for the most important variables (> ∣0.8∣) in PCA are in bold
